# Leveraging Nursing Management for Sustainable Healthcare: The Synergistic Impact of Green Human Resource Management, Sustainable Waste Management Practices, and Environmental Innovation

**DOI:** 10.1155/jonm/3381860

**Published:** 2026-06-28

**Authors:** Tipon Tanchangya, Diponkor Chandra Das, Farvez Islam, Abu Hena Md Martuza Ali, Kazi Omar Siddiqi

**Affiliations:** ^1^ Department of Finance, University of Chittagong, Chittagong, 4331, Bangladesh, cu.ac.bd; ^2^ Department of Management Studies, Comilla University, Kotbari, Comilla, 3506, Bangladesh, cou.ac.bd; ^3^ Department of Management, University of Chittagong, Chittagong, 4331, Bangladesh, cu.ac.bd; ^4^ Department of HRM, Bangladesh Open University, Gazipur, Bangladesh

**Keywords:** environmental innovation, green human resource management, sustainable, sustainable performance, the ability–motivation–opportunity theory

## Abstract

**Aim:**

This research explores the contribution of nursing management practices, particularly green human resource management (GHRM) and sustainable waste management (SWMP), to influence sustainable healthcare performance (SP) in hospitals in Bangladesh. The environmental innovation (EI) serves as a mediator. This study particularly focuses on nurse managers’ leadership in adopting these practices to enhance hospital sustainability outcomes.

**Design:**

A cross‐sectional quantitative study.

**Methods:**

We use purposive sampling to gather data. 388 nurses willingly participate in this work from five hospitals in Bangladesh. We also utilized validated Likert‐scale tools to assess GHRM, EI, and SP. The partial least squares structural equation modeling (PLS‐SEM) and necessary condition analysis (NCA) are employed to measure both direct and mediating effects.

**Results:**

The research finds that nursing management practices, particularly GHRM and SWMP, have a strong and significant impact on SP in healthcare. EI has a strong correlation with GHRM and SP. The outcomes suggest the crucial contribution of nurse managers in implementing these practices to increase sustainability outcomes.

**Conclusion:**

Nursing management practices, including targeted HRM strategies and waste management initiatives, are crucial for enhancing sustainable practices in healthcare. It is evident that nurse managers can play a key role in adopting and successfully applying these practices, which ultimately increase SP. Although GHRM has a direct correlation with SP, it has minimal influence via EI. Conversely, SWMP significantly benefits from nurse‐led EI, which ultimately improves sustainable healthcare outcomes.

**Implications for Nursing Management:**

Nurse managers have a crucial contribution to incorporating GHRM and SWMP into daily healthcare practices. Nurse managers can mitigate harmful environmental footprints through eco‐friendly innovation, sustainability training, and empowerment of nurses in decision‐making processes. These initiatives boost operational efficiency and assist in achieving the sustainable development goals (SDGs). These efforts also enhance patient care, particularly in resource‐limited settings.

## 1. Introduction

Global challenges such as environmental footprints, overusing resources, and pollution are driving a push for healthcare organizations to incorporate sustainability into their daily operations. Sustainability is now a major strategic priority in hospitals, influencing the quality of healthcare, patient safety, efficiency, and long‐term sustainability. Nurse leaders, in particular, play a key role as they represent the largest group of workers who support the patient care team in relation to waste, infection control, resource utilization, and patient care. They are in managerial positions that influence staff behavior, policies, and culture and have a direct impact on environmental outcomes and the effectiveness of sustainability initiatives. As primary stakeholders, nurses are responsible for caring for patients, managing resources, controlling infection, and segregation of waste. A robust healthcare system is crucial for delivering effective healthcare, economic stability, and environmental protection [1, 2]. By incorporating sustainability into operations, there is a higher chance of efficiency, less environmental impact, and high‐quality, long‐lasting services [3, 4].

Though the healthcare sector is vital for saving lives, it significantly impacts the environment due to excessive energy use, toxic and nonrecyclable waste, and carbon emissions. According to Sun et al. [5], this sector produces approximately 2.4 million tonnes of waste per year, which poses a significant threat to the environment. According to WHO data, about 15% of this waste is hazardous, such as pathological, radioactive, and infectious waste, and the remainder of waste, such as food, paper, and plastics, is municipal solid waste. Given these ecological challenges, sustainability is a critical issue for nurse leaders because waste management and unsustainable clinical practices have the potential to negatively affect patient safety, staff health, regulatory compliance, and healthcare quality. Nurse leaders have a very significant role in the implementation of sustainability at the ward level in nursing policies, daily routines, behaviors of nurses, and clinical procedures. Finally, achieving the goals of sustainability efforts will rely on the extent to which nurse managers can incorporate these policies into day‐to‐day nursing practices and staffing behaviors.

In Bangladesh, nursing management is a critical aspect, and the need for waste management and environmental protection is of utmost importance. The environmental management systems have shown insignificant improvement in Bangladesh, India, and Pakistan, as they are ranked 175th among 180 countries, according to the Environmental Performance Index [6]. Medical waste management in hospitals is still a great problem in Bangladesh, especially in the era of climate change and urbanization. While the political, governmental, and hospital sectors strive to improve waste management and mitigate climate risk, more actionable, nurse‐led solutions are needed to ensure sustainable healthcare and safeguard public health. The waste management system in Bangladesh, particularly in urban areas, is still poor: 55% of the solid waste goes uncollected, which consequently leads to pollution, health risks, and climate effects [7, 8]. The amount of medical waste keeps increasing at 3% per year, with nearly 25,000 tonnes being generated daily. Dhaka alone generates about 6500 tonnes per day, which is expected to grow to 8500 tonnes by 2032. The problems are poor infrastructure, lack of waste segregation, and poorly managed landfills. Dihan et al. [9] point out that limited research and a lack of data have led to the underwhelming focus of policymakers, authorities, media, and the public.

Instructing nurses with infectious and dangerous waste in clinical settings is a significant managerial and operational obstacle resulting from environmental deficiencies. Poor waste management increases the risk of hospital‐acquired infections, workplace hazards, and environmental pollution and reduces patient satisfaction. Furthermore, it is observed that there is a lack of skilled healthcare professionals, such as doctors, nurses, and technicians, particularly in government hospitals, with only 10% working on sustainable medical waste management [10]. Unlicensed waste disposal facilities frequently mix toxic medical waste with garbage, potentially compromising public health and environmental sustainability. These issues affect the credibility of institutions, patient satisfaction, and the quality of healthcare. Therefore, nurse leaders need to actively integrate sustainability into nursing practice, staff training, waste classification, and clinical governance [10, 11].

This study sees GHRM as a green nursing workforce management approach that addresses the problem issues of environment and operation in healthcare organizations. GHRM practices can be a new way to reduce the impacts of climate change and to build sustainable healthcare systems [12]. In a clinical setting, these practices are particularly relevant for nurse leaders, who are needed to train, motivate, evaluate, and support nurses to develop sustainable mindsets and environmentally responsible behaviors. Nurse leaders can bring in GHRM by strategies such as green recruitment, orientation programs based on sustainability, reward systems, sustainable training, and performance review systems that promote waste minimization and resource efficiency, support renewable energy, and ensure waste disposal. Although GHRM is known to support sustainability, there is limited evidence showing the direct effect of GHRM on sustainable performance in healthcare organizations [13, 14]. The current studies indicate that GHRM can enhance sustainable working practices, but there is little research on how it can be implemented in healthcare systems of emerging economies such as Bangladesh. This study, therefore, aims to explore the impact of GHRM on the management of the nursing workforce in the healthcare sector of Bangladesh.

Sustainable waste management practices (SWMPs) are important to nursing management as nurses contribute to waste segregation, infection control, waste disposal, and PPE management and follow waste‐management procedures. Nurse leaders will then be responsible for ensuring that clinical practice is carried out in an environment that is safe for them and minimizes risk of hazardous exposures in the hospital. While these practices can have a positive effect on environmental impact and resource efficiency, their impact on sustainable performance has not been fully explored. Most studies have been on the impact of SWMPs on cost‐effectiveness, institutional structures, community health, and environmental benefits such as pollution reduction [15]. In addition, most studies focus on operational or technological waste management, while there is a lack of attention to organizational and behavioral factors that play a key role in nursing leadership and long‐term sustainability [16, 17]. This gap is particularly significant in developing countries such as Bangladesh, where the quality of data, the enforcement of regulations, and the sustainability infrastructure are weak [18]. Recent studies also indicate that cultural innovation and behaviors toward sustainability significantly impact the connection between SWMPs and sustainable performance, but these aspects are under‐researched [19]. However, during the role of a nurse leader, policies alone will not guarantee the successful implementation of SWMP; nurse leaders also need to create sustainability training, ward‐level monitoring, and behavior change strategies to ensure that sustainability becomes a common part of the nursing day‐to‐day. Therefore, this study seeks to investigate the relationship between SWMPs and sustainable performance in the healthcare system in Bangladesh with a nursing management perspective.

Electronic innovation (EI) is a key element that helps shape nursing management in shaping sustainable work environments. It combines the use of technology with nurse‐led environmental projects like waste sorting, clinical care that promotes sustainability, the use of resources, and continuous quality improvement. Studies have shown that GHRM practices improve environmental responsibility and employee skills, which are crucial for EI and sustainable results [20]. However, there are still some unmet needs of understanding the extent and usage. Research evidence also suggests that there is limited and inconclusive evidence that there is a relationship between GHRM and EI, particularly in developing countries and healthcare settings [21]. The research is mostly conducted in industrial or corporate settings and has limited and inconclusive findings on EI specific to nursing management and healthcare sustainability [22]. It, therefore, emphasizes the importance of more empirical evidence and a comprehensive theoretical model that relates the GHRM, EI, and sustainable performance of nurses in Bangladesh.

SWMPs are essential to long‐term organizational sustainability, protection of the environment, and resource efficiency. Previous studies suggest that while SWMPs can have a positive impact on sustainable performance, they may not do so in isolation and may require organizational mechanisms to support their implementation [23]. Recent research indicates that EI, such as innovative waste reduction strategies, energy‐efficient processes, and eco‐friendly healthcare practices, can act as a mediator between SWMPs and sustainable performance [19]. However, there are still some gaps in the literature about the role of EI as a mediator in resource‐limited healthcare systems, particularly in developing countries such as Bangladesh [24]. The majority of existing research is centered on the industrial and corporate sector, with a lack of research in the healthcare and nursing management sector [18]. The gap is particularly important for nurse leaders, who are at the forefront of implementing sustainability initiatives at ward and unit levels and have a significant influence on nurses’ environmental actions through their leadership, supervision, and clinical governance. Therefore, examining the relationship between SWMP, SP, and GHRM and the role of EI as a mediator in the healthcare industry of Bangladesh can offer significant theoretical and practical knowledge about how nurse‐led sustainability initiatives can help to improve healthcare outcomes and environmental sustainability.

This study focuses specifically on nursing management rather than broader ecological management views. It explores the role of green nursing leadership and nurse‐led sustainability initiatives in influencing sustainable performance in hospitals in Bangladesh and how EI mediates the relationship. The study emphasizes that the strategic role nurse leaders can play in building sustainable cultures, promoting environmental accountability, strengthening clinical governance, and integrating sustainability into the activities of the hospital. As such, it is closely related to nursing leadership, operational management, workforce development, clinical governance, and implementation of sustainable healthcare practices in hospital environments.

## 2. Theoretical Background and Hypotheses Development

The resource‐based view (RBV) and the ability–motivation–opportunity (AMO) theories used in this study were first developed by Barney [25] and Appelbaum et al. [26] to assess the role of nursing management and nurse managers regarding sustainable healthcare practices. Both theories act as the main pillars of this study, transforming organizational ability into environmental practices and fostering SP. The RBV concentrates on developing products that are unique, noncomparable, and rare, as well as creating a competitive advantage in the market [25]. In nursing management, GHRM serves as a key contributor in healthcare organizations. The objectives of GHRM are employees’ ecological awareness and developing levels of confidence and skill, as well as promoting an eco‐friendly mindset. The combination of nursing leadership and GHRM creates a work environment that strives for green practices in regular clinical initiatives and ensures long‐term sustainability goals [25, 27]. According to RBV theory, GHRM enhances resource efficiency, reduces operational costs, and ultimately promotes SP [28]. These initiatives are crucial in nursing, where most of the tasks are human‐oriented and difficult to replicate. Moreover, GHRM serves as a key resource for operational success. Nurse managers can incorporate GHRM, develop sustainable nursing behaviors, and mitigate waste reduction, power consumption, and resource management [29]. When GHRM incorporates nursing practices, the sustainable work environment significantly increases in hospital sectors [30].

Similarly, AMO theory states that when nurses have the ability (knowledge & skills), opportunity (participation & involvement), and motivation (rewards & incentives) to perform, their sustainable mindset enhances significantly [26]. Nurse managers are key contributors to promoting this framework through offering green training, which empowers nurses to make ecological decisions and inspires green practices with incentives as well as rewards. These initiatives ultimately boost sustainability performance. In environmental sustainability in the clinical sector, human interaction is more crucial than technology, so strategies incorporating GHRM would be sufficient to boost sustainable performance [31].

The RBV model offers an effective framework for assessing the correlation of SWMP and SP in the healthcare sector. SWMP provides proper guidance to nursing managers’ leadership for introducing green practices [27]. When a hospital is able to implement such initiatives successfully, waste costs reduce significantly, and patient as well as staff safety levels increase notably. The responsibility of nurse managers is to adopt these practices into daily nursing routines and create innovative waste management systems that promote sustainability [29]. When nursing leadership incorporates SWMP in their strategies, it offers a durable sustainability advantage. Moreover, the AMO theory emphasizes nurse managers’ role in fostering innovation in hospital waste management systems and sustainability. When nursing leadership adopts innovative waste systems, it develops a sustainable mindset and motivates nurses to act in an eco‐friendly and innovative way. This framework also promotes sustainable innovations, including quality improvement, ecological awareness, and power conservation initiatives [30]. In healthcare, EI acts as a mediator within the nexus of SWMP and SP, where nurse managers play a key role in improving waste management systems. Correia et al. [29] found that nurse‐led innovations are crucial for achieving sustainable goals.

Finally, this study employs RVB and AMO theories in a nursing management context to explore how nurse managers promote sustainability through GHRM, SWMP, and EI in hospital sectors. The outcomes of this study highlight that nurse managers play a crucial role in developing a sustainable work environment in the healthcare sector. Furthermore, nursing leadership incorporating GHRM and SWMP develops a unique organizational culture that ensures healthcare efficiency and sustainable behavior in hospitals.

### 2.1. Green Human Resource Management (GHRM) and Sustainable Performance (SP)

GHRM refers to responsible HRM strategies that reduce an enterprise’s ecological footprint and encourage environmentally friendly practices. Prior studies have indicated that HRM activities improve organizational performance [32], although the majority of papers emphasize personal practices instead of the overall impacts of consolidated GHRM approaches. This establishes a vacuum in knowing how various HRM procedures collaborate to meet sustainable goals. In a similar way, Mousa and Othman [33] discovered that GHRM enhances staff wellness and health; however, they struggled to empirically connect these human resource advantages to overall organizational long‐term performance, particularly in healthcare settings. Furthermore, the majority of GHRM research is conducted in industrialized nations, making its relevance in developing nations such as Bangladesh poorly recognized. Applying the RBV and AMO models, this research investigates how combined GHRM procedures function as proactive, difficult‐to‐copy instruments that impact personnel’ capacities, inspiration, and chances to engage in more sustainable behaviors [26, 27]. This provides a greater understanding of how GHRM influences SP, with implications for theory and practice, especially in the unexplored medical field. Based on these findings, we offer the following hypothesis:

 H_1_ GHRM has a significant and positive effect on SP.

### 2.2. SWMP and SP

Medical waste has grown into a serious worldwide challenge, harming both the natural world and the general public [34]. Previous studies have looked at operating components of sustainable waste management (SWM), including energy consumption, employee abilities, discarding waste, conveyance, and care [35, 36]. However, those investigations primarily highlight SWM methods rather than explicitly tying them to quantified sustainable results. The World Health Organization (WHO) recommendations emphasize responsible disposal techniques and financial support, but they offer few insights into the behavioral and organizational elements that enable efficient execution. Proper SWMP implementation, which includes separation of waste, recycling, disposal safely, and resource‐saving ways, minimizes environmental contamination, minimizes health hazards, and increases hospital effectiveness, resulting in greater performance [37]. Clinics are complicated organizations whose sustainability is dependent not just on waste disposal policies but furthermore on personnel compliance, managerial backing, and organizational capability [29]. Recent studies indicate that clinics that integrate rigorous SWMP with employee instruction and tracking operate better ecologically and economically than those that use unstructured methods [38]. It is evident that when SWMPs are utilized properly, they enhance long‐term sustainable progress in hospitals. Utilizing AMO, this research pays more attention to the importance of staff quality, motivation, and involvement in using SWMPs for enhancing SP. This work addresses a critical vacuum by exploring how SWMPs influence SP in hospitals. It also provides theoretical and practical insights for medical sustainability. Relying on the preceding considerations, the following hypothesis is proposed:

 H_2_ SWMPs have a significant and positive effect on SP.

### 2.3. GHRM Practices, Environmental Innovation (EI), and SP

There are significant developments in economic and ecological outcomes worldwide, where medical institutions employ EI [39, 40]. Earlier studies suggest that GHRM promotes EI by cultivating a trained, ecologically aware labor force [3, 41], but few studies have thoroughly explored how EI acts as a mediator between GHRM practices and SP, especially in hospitals. According to the RBV theory, EI is an essential, nonimitable resource for the healthcare industry that enhances operational effectiveness and economic progress. Similarly, the AMO model focuses on how nurse managers can make an effective workforce and adopt green technologies. This work addresses a critical research gap by exploring how GHRM‐driven EI increases SP. It also suggests some crucial theoretical knowledge and recommendations for eco‐friendly medical administration. Thus, the following theory is proposed:

 H_3_ EI mediates the relationship between GHRM and SP.

### 2.4. SWMP, EI, and SP

Managing infectious healthcare waste (HCW) creates ecological and logistical hazards for medical facilities [42, 43]. Latest research explores clinical garbage disposal techniques and EI [3, 44, 45]. However, there is a paucity of research where EI serves as a mediator within SWMP and SP in hospitals. EI affects medical waste management methods via technology design, waste mitigation measures, and energy‐saving architecture [46], but the operational mechanisms that underpin these changes are unclear. According to RBV theory, EI is a tactical asset that can increase competitiveness and assist SP [21]. This study addresses a critical gap through empirically exploring whether EI mediates the association between SWMP and SP in Bangladeshi medical centers, offering theoretical understandings as well as actionable suggestions for long‐term healthcare governance. Based on the arguments presented above, the following hypothesis is proposed:

 H_4_ EI mediates the relationship between SWMP and SP.

### 2.5. Research Framework

**FIGURE 1:**
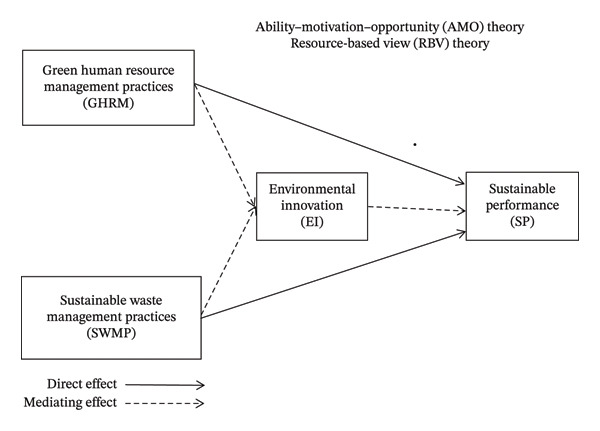
Research framework.

The research framework shown in Figure 1 was developed to illustrate the relationships among GHRM, SWMP, EI, and SP. This model is based on the AMO and RBV theories.

## 3. Methods

### 3.1. Sample and Procedure

**FIGURE 2:**
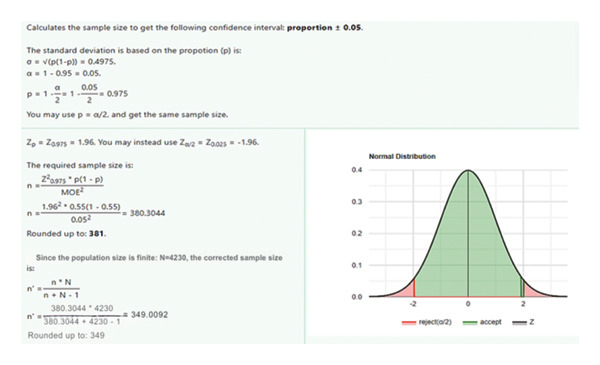
Calculation of sample size.

This research used established theories to develop hypotheses. As a result, a positivist approach was more appropriate in this study. This study also utilizes quantitative surveys for examining the relationship between variables and generalizing findings [47]. This study also utilizes a cross‐sectional approach for collecting data from nurses (including nurse managers) at five popular hospitals in Bangladesh. The five hospitals were chosen due to the homogeneity of their nursing staff. Rozario et al. [48] aimed to evaluate nurses’ roles in inpatient care at the hospital level in Bangladesh. Table 1 displays the number of nurses across the five hospitals, and Table 2 outlines the sampling procedure. Wolf et al. [49] recommend a minimum sample size of 300 for structural equation modeling (SEM). To ensure sufficient statistical power, we performed a formal sample size calculation using the *Z*‐score method for proportion estimation, which indicated that a sample size of 349 participants was necessary (see Figure 2). In the end, we gathered data from 388 participants to strengthen the robustness and generalizability of our results, exceeding the minimum sample size.

 Analysis: sample size
 Input: Tail (s) = Two
 Total population = 4230
 
*α* err prob. = 0.05
 Standard deviation is based on the proportion (*p*), which is 0.4975
 
*σ* = √(*p* (1 − *p*)) = 0.4975.
 
*α* = 1 − 0.95 = 0.05.
 Output: *Zp* = *Z*0.975 = 1.96; we may instead use *Zα*/2 = *Z*0.025 = −1.96.
 Total sample size = 349 (minimum)

**TABLE 1:** Number of nurses in five selected hospitals in Bangladesh.

Serial no.	Hospital name	No. of nurses	Reference
*Government hospital*			
1	Dhaka Medical College Hospital, Dhaka	2275	Facility Registry (Government of People’s Republic of Bangladesh, Ministry of Health and Family Welfare) [50]
2	Mymensingh Medical College Hospital, Mymensingh	1029	Facility Registry (Government of People’s Republic of Bangladesh, Ministry of Health and Family Welfare) [51]
3	Cumilla Medical College Hospital, Cumilla	416	Facility Registry (Government of People’s Republic of Bangladesh, Ministry of Health and Family Welfare) [52]

*Private hospital*			
4	Comilla Medical Center (Pvt.) Ltd.	252	Hospital Source
5	Moon Hospital Ltd.	258	Hospital Source

Source: [49–51].

**TABLE 2:** Selected sample from five hospitals and sampling procedure.

Serial no.	Hospital name	No. of nurses (population)	Sample
*Government hospital*			
1	Dhaka Medical College Hospital, Dhaka	2275	155
2	Mymensingh Medical College Hospital, Mymensingh	1029	93
3	Cumilla Medical College Hospital, Cumilla	416	52

*Private hospital*			
4	Comilla Medical Center (Pvt.) Ltd.	252	45
5	Moon Hospital Ltd.	258	43
	Total	4230	388

Source: Author’s self‐estimation.

The respondents of this study are nurses (nurse managers) with more than 3 months of professional license. They are also current service persons of private and government‐owned hospitals. In Bangladesh, Roy et al. [53] found that using the existing list of nurses and applying the random sampling method is not feasible for the following reasons: most of the nurses change their workplace very often, and those nurses who work in public hospitals also work in private hospitals part‐time. Therefore, using random sampling might result in duplicating public healthcare workers. Consequently, this study employed nonprobabilistic purposive sampling. This study used a cross‐sectional approach, collecting data from nurses to meet its research goals. Nurses were chosen as the only respondents because they are directly involved in patient care and hospital operations, making them the best source of knowledge about safety, health, and environmental practices [54]. The goal was to analyze the SP of medical centers, and a purposeful sampling approach was adopted. This strategy prioritized nurses who were engaged in environmentally friendly activities, as opposed to random sampling, which may have contained nurses with an inadequate understanding and so harmed information quality. Purposive sampling increased the significance and precision of the outcomes. We acknowledge that nonprobability sampling may lead to biased selection because of the investigator’s discretion in choosing participants. To deal with this, nurses were chosen from five medical institutions of varying sizes and areas to acquire various perspectives and eliminate biased responses.

Before collecting data, all respondents were provided with clear details regarding the purpose of the study, methods, and their rights, such as the voluntary aspect of engagement and the possibility of withdrawal. Because data were acquired using self‐reported questionnaires, several methodological and statistical procedures were used to avoid possible biases. Participants were guaranteed anonymity and confidentiality to minimize social desirability bias [55]. The survey items, adapted from validated scales, were pilot‐tested and neutrally phrased to prevent leading responses. Additionally, common method bias was evaluated using the full collinearity approach [56].

According to Siddiqi et al. [57], a sample size of 300 or more is enough for SEM. The sampling procedure also followed the formula for the known population, yielding an estimated minimum sample size of 349. This research used the drop‐off/pick‐up (DOPU) data collection technique. Respondents share information willingly without any incentives. The researchers distributed the questionnaires to hospital administrators, who then handed out the hard copies to nurses. A total of 1000 questionnaires were distributed across five hospitals. The researchers followed up with hospital administrators via phone calls throughout the process. Nurses returned their completed questionnaires to their hospital administrators. The respondent had 2 months to share information, and then, the author collected the questionnaire from hospital administrators. Ultimately, data were collected from 418 Bangladeshi nurses between June 2025 and July 2025. After removing responses with missing data, 388 responses remained (as shown in Figure 2) and were used for the final data analysis. The average response rate was 38.80%. The 38.8% response rate was evaluated by comparing respondent demographics with available population data, suggesting minimal nonresponse bias.

### 3.2. Measurements

In this study, the existing resources were utilized in stating the survey tools. All the items of the GHRM, SWMPs, EI, and SP variables were assessed through a Likert‐type scale that primarily varies between (1) denoting utmost disagreement and (5) representing maximum agreement. The study used the back‐translation method, as stated by Liao et al. [58]. The original questionnaire was in English. We translated all English items into Bangla and then back to English, following the recommendations of Liao et al. [58], to ensure content clarity of the measuring instruments. The questionnaire was refined through two pilot test rounds with 25 and 30 participants, respectively. The primary aim of piloting was to identify any unclear items in Bangla. Feedback from the first round revealed that some items were ambiguous, so we revised these to improve their clarity. The second pilot round confirmed that the questionnaire was fully clear. A satisfactory level of adequate reliability (*α* > 0.70) is found in all scales [59]. In examining the exogenous variable GHRM, a 5‐item scale was used, as indicated by Dumont et al. [60]. Furthermore, to analyze SWMPs, the 5‐item scale developed by Quartey et al. [61] was utilized. In addition, to analyze SP, the 8‐item scale developed by Paille et al. [62] was employed. To analyze EI as a mediator, a 6‐item scale developed by Wong et al. [63] was employed.

### 3.3. Data Analysis Strategy

This study primarily utilized SPSS statistical software to analyze the data, collect respondents’ demographic details, and generate descriptive statistics. Additionally, the formulated hypotheses were examined using the PLS‐SEM approach. According to Dash and Paul [64], PLS‐SEM serves both predictive and explanatory functions and is suitable for inductive and formative models. To uphold thorough logical reasoning, necessary condition analysis (NCA), developed by Dul [65], was also employed.

### 3.4. Methodological Inconsistency

Before data analysis, the authors of this study ensured common method bias through proper investigation. We applied two variants of the PLS‐SEM model in our study using SmartPLS [59]: the full collinearity approach and the variance inflation factor (VIF) [56]. Our research revealed that VIF values were consistently less than 3.3 [59] (refer to Table 3), reflecting the absence of bias in the research data.

**TABLE 3:** Common method variance/bias.

Construct	EI	GHRM	SP	SWMP
Environmental innovation (EI)			1.10	
Green human resource management (GHRM)	1.01		1.02	
Sustainable performance (SP)				
Sustainable waste management practices (SWMPs)	1.01		1.10	

### 3.5. Profile of the Respondents

Table 4 represents the demographic profile of the respondents. This demographic profile includes different characteristics of the respondents, such as gender, age, educational qualification, service period, professional position, marital status, and religion. Most of the respondents were female (85.31%), while male respondents were only 14.69%. Moreover, the majority of participants are in the young age range within 30 years (46.91%), followed by those aged 31 to 40 years (25.77%), 41 to 50 years (18.81%), and over 50 years (8.51%). The majority of participants (67.27%) hold a diploma degree, followed by a bachelor’s (15.21%) and a master’s (13.14%) degree in nursing. Almost all majority respondents have more than 5 years of work experience in the healthcare industry. Additionally, 30.41% of nurses at the current hospital have worked there for more than 3 months but no more than 2 years. 17% of nurses have two to 3 years of work experience, and 14.95% have four to 5 years of practical experience. In terms of employment, the highest percentage of participants (75.52%) works in the public sector, while 24.48% of participants work in the private sector. Most nurses (82.22%) are Muslim, followed by 13.40% who are Hindu and 4.38% from other faiths. Overall, 28.61% of respondents were single over the 5 years, while the majority (71.39%) were married.

**TABLE 4:** Profile of respondents.

		Frequency	Percent
Gender	Male	57	14.69
	Female	331	85.31

Age	30 and below	182	46.91
	31–40 years	100	25.77
	41–50 years	73	18.81
	Above 50 years	33	8.51

Highest education	Secondary school certificate	8	2.06
	Higher secondary certificate	9	2.32
	Diploma in nursing	261	67.27
	Bachelor in nursing	59	15.21
	Master in nursing	51	13.14

Length of service	More than 3 months but less than 2 years	118	30.41
	2‐3 years	60	15.46
	4‐5 years	58	14.95
	More than 5 years	152	39.18

Employment	Public sector	293	75.52
	Private sector	95	24.48

Religion	Islam	319	82.22
	Hindu	52	13.40
	Others	17	4.38

Marital status	Married	277	71.39
	Unmarried	111	28.61

### 3.6. Descriptive Statistics

According to Ho [66], the following explanation for mean scores indicates that the range 1–2.33 means low, 2.34–3.66 means moderate, and 3.67–5 represents high. According to Table 5, SWMP has a moderate mean, and all other constructs demonstrate high means.

**TABLE 5:** Descriptive statistics.

Descriptive statistics			
Construct	*N*	Mean	Std. deviation
	Statistic	Statistic	Statistic
Green human resource management (GHRM)	388	3.83	1.08
Sustainable waste management practices (SWMPs)	388	3.50	1.22
Environmental innovation (EI)	388	3.78	1.09
Sustainable performance (SP)	388	3.71	1.03
Valid *N* (listwise)	388		

### 3.7. Assessing Measurement Model

**FIGURE 3:**
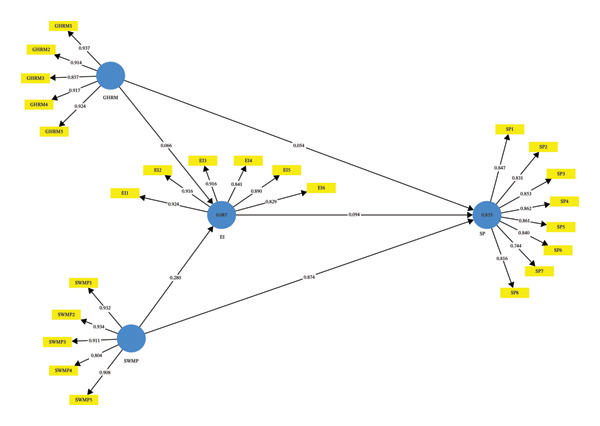
Measurement model.

In this study, we utilized a version of SmartPLS 4 (4.1.0.2) to assess all the hypotheses. According to Hair et al. [59], the standard value of a Cronbach’s alpha is 0.70, while 0.80 to 0.90 is also preferable. In Table 6, the value of all Cronbach’s alphas exceeded the threshold, indicating a strong internal consistency. Hair et al. [59] also proposed that the standard value of composite reliability is more than 0.70. In this study, all composite reliability values are more than the standard level, indicating strong composite reliability. We employed outer loadings and AVE to test convergent validity. The standard loadings value must exceed 0.70 [59]. This basically indicates that a variation of more than 50% in the indicator is acceptable, ensuring reliability. According to Table 6, all outside loading values are more than 0.70. Furthermore, the standard value of AVEs has to be more than 0.50 for convergent validity [67]. This study found that all AVEs range from 0.69 to 0.83, indicating that there are no issues regarding convergent validity. Figure 3 also confirms the validity and reliability issues of the study.

**TABLE 6:** Test of internal consistency and convergent validity.

Constructs	Items	Outer loadings	*Cα*	CR (rho_a)	CR (rho_c)	AVE
Green human resource management (GGRM)			0.95	0.95	0.95	0.83
	GHRM1	0.94				
	GHRM2	0.91				
	GHRM3	0.86				
	GHRM4	0.92				
	GHRM5	0.92				

Sustainable waste management practices (SWMPs)			0.94	0.94	0.95	0.81
	SWMP1	0.93				
	SWMP2	0.93				
	SWMP3	0.91				
	SWMP4	0.80				
	SWMP5	0.91				

Environmental innovation (EI)			0.95	0.95	0.95	0.79
	EI1	0.92				
	EI2	0.92				
	EI3	0.92				
	EI4	0.84				
	EI5	0.89				
	EI6	0.83				

Sustainable performance (SP)			0.94	0.95	0.95	0.69
	SP1	0.85				
	SP2	0.83				
	SP3	0.85				
	SP4	0.86				
	SP5	0.86				
	SP6	0.84				
	SP7	0.74				
	SP8	0.82				

We test discriminant validity in Tables 7 and 8 by utilizing the Fornell–Larcker criterion and Heterotrait–Monotrait ratio (HTMT). In Table 7, the diagonal constructs’ values are higher than the off‐diagonal constructs, indicating strong discriminant validity [68]. Hair et al. [67] suggest that the standard value of HTMT should be less than 0.90 and all values of Table 7 should be below the maximum level, so all constructs validate discriminant validity.

**TABLE 7:** Results of Fornell–Larcker criteria.

Constructs	EI	GHRM	SP	SWMP
Environmental innovation (EI)	0.89			
Green human resource management (GHRM)	0.10	0.91		
Sustainable performance (SP)	0.35	0.16	0.91	
Sustainable waste management practices (SWMPs)	0.29	0.11	0.83	0.90

**TABLE 8:** Results of HTMT.

Constructs	EI	GHRM	SP	SWMP
Environmental innovation (EI)				
Green human resource management (GHRM)	0.09			
Sustainable performance (SP)	0.37	0.17		
Sustainable waste management practices (SWMPs)	0.30	0.11	0.83	

### 3.8. Assessing Structural Model

**FIGURE 4:**
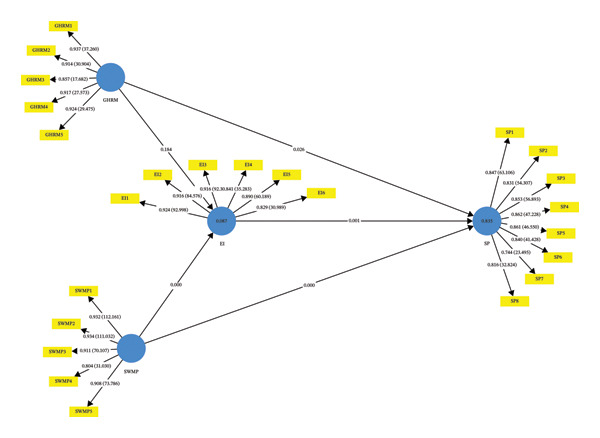
Structural model.

The structure model of this work is demonstrated in Figure 4. We use 10,000 bootstrap subsamples with a two‐tailed test for identifying the bias‐corrected percentile approach [67].

According to Table 9, the R2 value of 0.84 for sustainable performance indicates that the independent variables included in the model collectively explain 84% of the variance in sustainable performance among the healthcare settings. This high explanatory power suggests that the model has substantial predictive capability and that the selected predictors are strongly associated with sustainable performance outcomes. Hair et al. [59] suggested that *R* ^2^ value above 0.75 is considered substantial, highlighting the robustness of the model in capturing the key determinants of sustainable performance [59]. Additionally, a *Q* ^2^ predict value of 0.82 for sustainable performance shows that the model has strong predictive power. This means it can reliably forecast out‐of‐sample observations related to sustainable performance, highlighting its robustness [59]. According to Hair et al. [67], a *Q* ^2^ predict value above 0.35 demonstrates substantial predictive relevance, suggesting that the model’s variables provide valuable insights into predicting sustainability outcomes in healthcare. This finding emphasizes the model’s practical usefulness for forecasting sustainability outcomes and ensures the essential key determinants from both theoretical and managerial points of view.

**TABLE 9:** Results of the coefficient of determination (*R* ^2^) and predictive relevance (*Q* ^2^).

	*R*‐square	*R*‐square adjusted	*Q* ^2^ predict	RMSE	MAE
Sustainable performance (SP)	0.84	0.83	0.82	0.42	0.30

Following the guidelines of Henseler et al. [69] and Rahman et al. [7], the standardized root mean squared residual (SRMR) was used to assess the model’s goodness‐of‐fit. Consistent with Hair et al. [70], SRMR values under 0.08 suggest an acceptable fit. In our study, the SRMR was 0.054, indicating a good model fit (see Table 10). We also applied the Bentler–Bonett Normed Fit Index (NFI) to evaluate the model’s approximate fit [69]. Singh [71] states that NFI values between 0.60 and 0.90 are acceptable. The NFI in this study was 0.851 (Table 10), supporting the model’s adequacy.

**TABLE 10:** Results of model fitness.

	Saturated model	Estimated model
SRMR	0.054	0.054
d_ULS	0.862	0.862
d_G	0.687	0.687
Chi‐square	1535.495	1535.495
NFI	0.851	0.851

**FIGURE 5:**
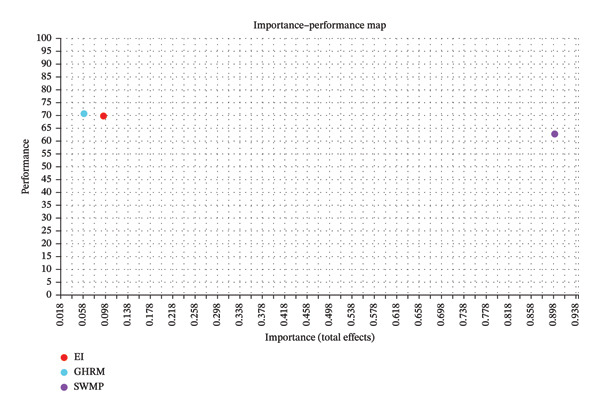
Importance–performance map.

The IPMA results reveal (Table 11 and Figure 5) the relative importance and performance of various latent variables affecting SP. SWMPs have the most significant total effect (0.90) on SP, marking them as the key driver of sustainable performance. However, their performance index (62.62) indicates considerable potential for enhancement. In contrast, EI and GHRM show lower total effects (0.09 and 0.06, respectively) but higher performance indices (69.70 and 70.56), implying that they perform relatively well but have a modest impact on SP. These insights suggest that focusing on improving SWMPs—such as better waste segregation, recycling, and eco‐friendly disposal—could lead to the most substantial improvements in sustainable performance within healthcare. Meanwhile, ongoing enhancement of EI and GHRM practices can sustain and support long‐term sustainability by capitalizing on their current strengths.

**TABLE 11:** IPMA results full data set.

Sustainable performance (SP)		
Latent variable	Total effect (importance)	Index value (performance)
Green human resource management (GHRM)	0.06	70.56
Sustainable waste management practices (SWMPs)	0.90	62.62
Environmental innovation (EI)	0.09	69.70

We employed bootstrapping to assess the construct’s path coefficient. According to Hair et al. [59], the reliable value of *p* value is less than 0.05, while it is deemed 1.96 for the critical value for *T*‐statistics of a two‐tailed test. The writers also suggested that the path coefficient is significant at the 0.05 level if the 95% confidence interval excludes 0. In Table 12, the path analysis results show that both GHRM and SWMP positively affect SP, though to different degrees. GHRM has a small but statistically significant direct impact on SP (*β* = 0.05, *t* = 2.22, *p* = 0.03, 95% CI [0.01, 0.10]), suggesting that green HR initiatives can slightly boost organizational sustainability. Meanwhile, SWMP has a strong, highly significant direct effect (*β* = 0.87, *t* = 62.09, *p* < 0.05, 95% CI [0.85, 0.90]), underscoring the crucial role of waste management in enhancing sustainable performance. As for mediation, EI does not significantly mediate the GHRM‐SP link (*β* = 0.01, *t* = 1.16, *p* = 0.25, 95% CI [−0.00, 0.02]), indicating that GHRM influences sustainability mainly through direct effects. However, EI significantly mediates the SWMP‐SP relationship (*β* = 0.03, *t* = 2.76, *p* = 0.01, 95% CI [0.01, 0.05]), indicating that the positive impact of waste management on sustainability partly operates via environmental innovation. Ultimately, these findings highlight the dominant direct and indirect impacts of SWMP on organizational sustainability, while GHRM’s effect remains more modest and direct.

**TABLE 12:** Results of path coefficient and confidence interval test.

Hypothesis	Path coefficient				Confidence interval		
	Path	Original sample (O)	*T* statistics	*p* values	5%	95%	Decision
*Direct effect*							
H_1_	GHRM ⟶ SP	0.05	2.22	< 0.001	0.01	0.10	Supported
H_2_	SWMP ⟶ SP	0.87	62.09	< 0.001	0.85	0.90	Supported

*Mediation effect*							
H_3_	GHRM ⟶ EI ⟶ SP	0.01	1.16	0.250	−0.00	0.02	Not supported
H_4_	SWMP ⟶ EI ⟶ SP	0.03	2.76	< 0.001	0.01	0.05	Supported

### 3.9. NCA

**FIGURE 6:**
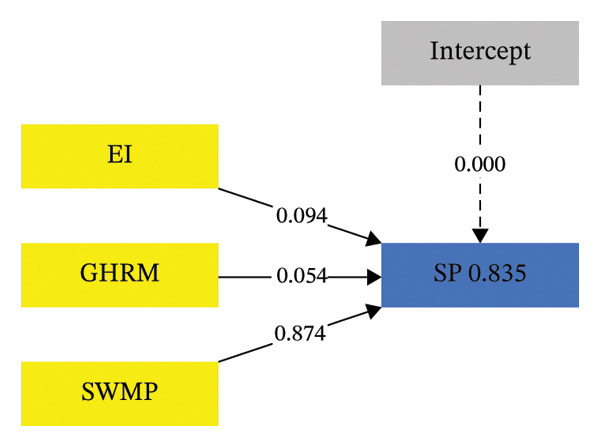
Ceiling line effect size.

Furthermore, the correlation between exogenous variables (GHRM, SWMP, and EI) and SP is analyzed by utilizing NCA. Dul developed the NCA method in 2016 and combined it into SmartPLS V4. Dul [65] suggested that a small, medium, large, and very large effect size is when the NCA values are 0 < *d* < 0.1, 0.1 ≤ *d* < 0.3, 0.3 ≤ *d* < 0.5, and *d* ≥ 0.5, respectively. Dul et al. [72] also proposed to use an “approximate permutation test” to assess the evidence against the null hypothesis. This test helps us to find out the exact *p* value and reduce Type 1 errors and false‐positive conclusions. According to Table 13 and Figure 6, the effect size of this work ranges from 0.05 to 0.87, indicating minimal effects of GHRM and EI on GI. SWMP has a strong and significant impact on predictor variables on SP. The permutation test with *p* values also confirmed that the effect size of the variables is within the standard. All the permutation values are significant (*p* < 0.05).

**TABLE 13:** Ceiling line effect size (CE‐FDH).

Constructs	Original effect size	95%	Permutation *p* value
Green human resource management (GHRM)	0.05	0.03	< 0.001
Sustainable waste management practices (SWMPs)	0.87	0.01	< 0.001
Environmental innovation (EI)	0.09	0.02	< 0.001

**FIGURE 7:**
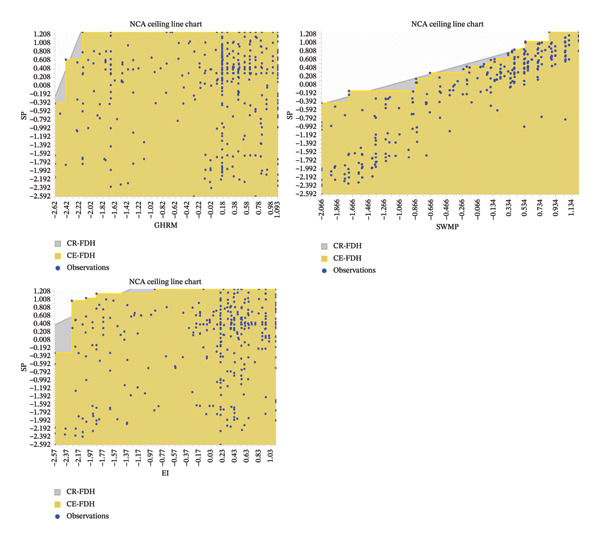
NCA ceiling line chart.

The suggested ceiling line is used to condone signaling extraneous linear assumptions in predictors with outcome variables. According to Dul [65], there are two different ceiling lines, such as ceiling envelopment–free disposal hull (CE‐FDH) as well as ceiling regression–free disposal hull (CR‐FDH). In terms of application, the CE‐FDH is employed for discrete data or data with a few levels, while the CR‐FDH is employed for data that are changing continuously. Our data are best suited for the CE‐FDH line. Figure 7 demonstrates that the ceiling line indicates a particular level of SP required to obtain a certain level of endogenous variable [73].

The combined analysis of PLS‐SEM and NCA indicates that SWMP and GHRM serve distinct but complementary roles in enhancing sustainable performance (see Table 14). SWMP has a strong, significant direct influence on SP (*β* = 0.87, *p* < 0.05) and is identified as both a sufficient and necessary condition (*d* = 0.87, *p* < 0.05), highlighting its central role as a primary driver and prerequisite for superior sustainability outcomes.

**TABLE 14:** Necessity and sufficiency results—sustainable performance.

PLS‐SEM—significance analysis path				Necessary condition analysis (NCA)			
Hypothesis	Path coefficient	*p* value	Sufficiency	Path	Effect size (*d* value)	*p* value	Necessary condition
GHRM ⟶ SP	0.05	< 0.001	Fulfilled	GHRM ⟶ SP	0.05	< 0.001	Fulfilled
SWMP ⟶ SP	0.87	< 0.001	Fulfilled	SWMP ⟶ SP	0.87	< 0.001	Fulfilled
GHRM ⟶ EI ⟶ SP	0.01	0.250	Not fulfilled	EI ⟶ SP	0.09	< 0.001	Fulfilled
SWMP ⟶ EI ⟶ SP	0.03	< 0.001	Fulfilled				

GHRM has a smaller but significant direct influence (*β* = 0.05, *p* = 0.01) and has a necessary condition (*d* = 0.05, *p* = 0.01). These statistical results ensure that GHRM practices are crucial for achieving high performance, but other factors have to be taken into consideration. In terms of mediation, GI positively mediates SWMP’s effect on SP (*β* = 0.03, *p* = 0.01) and is necessary for achieving high SP (*d* = 0.09, *p* < 0.05), but GHRM’s indirect effect via EI is insignificant. These results emphasize that institutions should pay attention to SWMP and promote environmental innovation as key strategies, while continuing to uphold fundamental GHRM practices, to ensure these factors are both sufficient and necessary to effectively drive sustainable performance.

## 4. Discussion

This work revealed a strong and positive correlation between GHRM and SP (H1). It also emphasizes the key role of nurse managers in advancing GHRM practices and how it influences SP in hospitals’ operations. It is the responsibility of the nurse manager as a key leader to develop GHRM strategies into nursing operations as well as create a conducive working environment that fosters sustainability. They can make their working environment environmentally friendly through introducing green recruitment, training, and performance management programs. Such activities will inspire nurses to adopt a sustainable nursing mindset [74, 75]. Furthermore, they can apply sustainable practices like waste segregation and energy‐efficient resource use and develop environmental protocols that significantly upgrade the level of SP. The application of AMO theory in this work shows how nurse managers can upgrade nurses’ ability, opportunities, and motivation to embrace a sustainable mindset and emphasize GHRM practices for superior SP [76]. Jnaneswar [75] also investigated that when management adopts GHRM in their core corporate strategies, it enhances sustainable practices as well as the hospital’s reputation. This work ultimately emphasizes the contribution of nurse managers to promote a sustainable‐oriented healthcare environment by utilizing effective leadership, corporate strategies, and a sustainable mindset to address environmental hazards. It also offers valuable insights for concerned authorities to adopt a practical framework to integrate sustainable practices in daily routines and healthcare management.

This study also finds a significant correlation between SWMP and SP (H2). This work particularly focuses on the crucial contribution of nurse managers in effectively adopting SWMP and how it strives for sustainable growth in the healthcare industry. As a concerned authority, nurse managers can play a crucial role in integrating eco‐friendly initiatives into daily nursing activities, including waste segregation and safe sharps disposal, as well as recycling nonharmful materials. These practices ultimately minimize harmful footprints, enhance safety, and ensure sustainable standards in healthcare activities. Such activities also promote sustainable healthcare, including minimizing environmentally harmful footprints, preserving resources, and creating awareness regarding community health [61, 77]. Moreover, the results of this study emphasize the key role of nurse managers to promote sustainability through training, support, and a conducive work environment to own sustainable waste practices. Nurse managers can develop GHRM strategies in their core corporate strategies so that nurses develop a sustainable mindset, behave in an environmentally responsible way, and focus on better sustainable outcomes. This study also highlights that nurse managers have to integrate SWMPs in their strategies in order to develop durable healthcare settings.

This study also found an insignificant correlation between GHRM and SP in the context of the mediating role of EI (H3). This could happen due to different factors, including organizational strategy, leadership, and resource availability [78]. Nurse managers are the key mechanism here to connecting GHRM practices with environmental innovation in the healthcare sector. They can foster green practices, GI, and a sustainable‐oriented work environment to enhance sustainable outcomes. Yong et al. [79] investigated that some institutions represent GHRM to promote their brand but occasionally lack innovation. However, nurse managers can foster GI through training, active engagement, and effective sustainability efforts. This approach assists nurse managers in increasing both sustainable practices and environmental innovation at the ward level. Proper utilization of green training, leadership, and GHRM, nurse managers can change organizational behavior. This study found that nurse managers are the key contributors to GHRM, and innovation is the key mechanism for sustainable healthcare performance. However, some external factors or organizational strategies may create obstacles to the full mediating potential of EI.

This study found that EI as a mediator has a strong and significant contribution to the correlation between SWMPs and SP (H4). Utilizing RBV and AMO theories, the outcomes from PLS‐SEM and NCA also confirmed H4. It is evident that EI has significantly mediated the nexus within SWMPs and SP, but nurse managers are the key contributors to promoting these healthcare innovations. Their roles are essential for both minimizing harmful environmental footprints and developing sustainable‐oriented working environments. According to Liu et al. [80] and AlSheyadi et al. [81], it was also argued that including zero‐waste and recycling initiatives in nursing practices encourages nurses to adopt green innovation at the ward level. These initiatives greatly reduce environmental impacts, such as closed‐loop systems and sustainability efforts. As statistical evidence affirms that SWMPs are crucial for increasing SP, nurse managers’ leadership has to include them in their daily routines, which truly strives for sustainability. This research highly emphasizes the contribution of nurse managers for enhancing sustainable healthcare, utilizing environmental innovations in nursing leadership and routine care.

This work finds that SWMPs have a significant influence on SP (*β* = 0.87) compared to GHRM (*β* = 0.05). Immediate actions, including waste segregation, disposal systems, and compliance, are more effective for sustainability than HR‐oriented practices in the Bangladeshi healthcare sector. These actions directly enhance environmental actions like waste management and resource conservation, which ultimately drive favorable patient care and hospital sustainability [82]. The mediating effect of EI on the GHRM and SP relationship was nonsignificant, indicating that EI does not significantly bridge GHRM and performance in this context. This may be a gap addressing the need for innovative practices within HR strategies. This may happen due to resource limitations or a lack of administrative strategies [83]. Since nursing management often controls HR practices, the weaker influence of GHRM emphasizes the need to prioritize SWMPs over purely HR‐focused initiatives to advance hospital sustainability. This aligns with recent research suggesting that nursing managers should focus not only on green HR practices such as training and policies but also on integrating practical sustainability measures into nursing workflows to improve both environmental and operational outcomes [84]. Ultimately, the results highlight the vital role of hospital administrators and nursing leaders in ensuring that practical, resource‐based initiatives, such as waste management, are valued alongside long‐term HR strategies to promote sustainable healthcare.

### 4.1. Theoretical Implications

From a theoretical point of view, this research supports all hypotheses except H3. This study supports both the RBV and AMO theories in the scope of environmental management in Bangladesh. In order to propose a strong conceptual model, this study used SEM and NCA tests. This study initially explores how GHRM’s influence on SP in the healthcare sector can make a significant contribution by developing the existing literature on HRM and eco‐management. It is evident that healthcare institutions have a significant contribution to environmental damage and climate change [85], particularly in Bangladesh, which remains an underexplored yet important context. Then, this paper did not support the mediating role of EI in the nexus between SWMPs and SP. As a result, this result is more unique than previous studies. So, the relationship between SWMPs and SP fills a crucial literature gap in sustainability theory and makes a valuable contribution to organizational performance. This contribution helps to develop a standard framework and fix gaps in the literature on environmental management, strategic management, and sustainability. SWMPs enhance SP and expand RBV by viewing waste management systems as strategic internal resources and skills that boost company competitiveness. This connection also advances AMO theory by leveraging human capabilities and shows the link between HRM and environmental practices. Finally, the current study adds to the existing literature by highlighting the mediating role of EI in the connection between SWMPs and SP. The mediating role of EI in our research has improved understanding of how management perceives and is inclined to engage in green business activities through SWMPs, thereby significantly enhancing the SP of healthcare industry concerns. This study is unique because of its exploration of the relationship between SWMPs and SP via EI, thereby expanding knowledge of unexplored outcomes related to renewable energy, water conservation, waste management, and eco‐friendly infrastructure from a sustainable performance perspective in the healthcare industry.

### 4.2. Implications for Nursing Management

This study offers nurse leaders practical and policy suggestions to improve hospital sustainability through the use of GHRM, SWMPs, and EI. Nurses have a pivotal position in the healthcare environment and play a role in clinical practice, staff conduct, patient safety, adherence to regulatory laws and norms, and organizational culture. In a world that is grappling with greater environmental issues, higher costs, and more complex sustainability requirements, nurse leaders must recognize sustainability as a key managerial and professional responsibility, not a nice‐to‐have program. They play a particularly important role since they are directly engaged in resource use, waste generation, and patient care processes that affect environmental outcomes and are the largest group of employees in hospitals. Nurse leaders need to create formal green training and continuing education for effective implementation of sustainability initiatives. These programs can provide education to nurses on sustainability in clinical practice, waste segregation, energy conservation, infection‐control sustainability, and safe disposal. This training can help increase nurses’ environmental awareness and improve compliance with SWMPs in their daily clinical practice. Furthermore, embedding sustainability objectives within the orientation, competency assessment, and leadership development processes of employees can contribute to building a culture of sustainability. Furthermore, some nurses have a poor understanding of the importance of environmental responsibility, and implementing a reward and recognition program can encourage them to take up environmentally responsible practices, such as minimizing unnecessary resource consumption, recycling, waste reduction, and actively participating in waste reduction initiatives.

Nurse leaders can introduce unit‐specific sustainability tracking and performance review systems to increase accountability and compliance with sustainability requirements. They can track adherence to waste management regulations, manage nurse‐led environmental projects, and incorporate sustainability metrics into performance assessments, among other tasks. These efforts can enhance compliance with environmental regulations, operational efficiency, and patient safety. Further, through the interdisciplinary sustainability committees, nurse leaders can help encourage nurses to be part of nurses’ quality improvement projects that are environmentally focused, creating innovation and shared responsibility for sustainable health care. Building a sustainable mindset among nurses can improve engagement, accountability, and making sustainable decisions. Nurses who are more aware of environmental issues are more likely to take proactive measures, think outside the box, and have a greater commitment to sustainable healthcare practices [86]. This can be achieved by nurse leaders who clearly communicate sustainability goals, incorporate them into team conversations, and set a positive example for sustainable leadership. Simple, sustainable changes to the way nursing teams use energy, reuse materials where possible, and eliminate unnecessary medical waste can make the healthcare environment more sustainable.

Institutional and policy support can also play a key role in the successful implementation of these initiatives. Healthcare policy, leadership, and national regulators must create appropriate policy frameworks, infrastructure, and resources to support sustainability initiatives by nurses. The Environmental Conservation Act and WHO’s hospital sustainability guidelines are important policies that support sustainable healthcare management in Bangladesh [87, 88]. Therefore, nurse leadership needs to collaborate with hospital administration and policy makers to establish evidence‐based sustainability policies for the hospital that support national environmental policies and hospital sustainability. These strategic leadership actions can make a substantial contribution to creating sustainable healthcare systems that provide quality patient care, environmental sustainability, and organizational resilience even under resource limitations.

### 4.3. Contribution to Society

As environmental issues are becoming a pressing issue, this study offers an approach to enhance environmental management, providing more benefits to society and the planet. The healthcare sector significantly generates environmentally toxic waste. An environmentally conscious society always seeks such hospitals, which concentrate on minimum levels of infectious and hazardous waste from their operations and develop a sustainable mindset that fosters resource efficiency. This study model and the findings of this study emphasize SP and its drivers, including GHRM, SWMP, and EI. If healthcare institutions in Bangladesh adopt this environmental management model, society will benefit from sustainable‐oriented initiatives, reduced levels of environmental degradation, enhanced resource efficiency, and a higher standard of living.

## 5. Conclusion, Limitations, and Future Research Scope

This empirical work was conducted in healthcare sectors in Bangladesh, a lower‐middle‐income country. The outcome of this research offers valuable information on the subject and fulfills a significant gap in previous sustainability studies. This study offers a new model that provides an in‐depth perspective of the GHRM and SWMP‐SP relationship by exploring the mediating role of EI. To verify the relationships, the PLS‐SEM and NCA tests were also used. The findings align with RBV and AMO theories, offering statistical evidence of the favorable, significant influence of GHRM and SWMP on SP via EI in the healthcare sector in Bangladesh. The outcomes reveal that GHRM and SWMP are significantly connected to SP. Additionally, EI mediates the correlation between SWMP and SP. However, the nexus between GHRM and SP via EI was insignificant.

This study examined only the healthcare sector in Bangladesh, a country that is one of the contributors to ecological hazards. Due to its limited scope as well as sample size, the findings’ generalizability is restricted. Future studies must involve other sectors and bigger, more diverse samples to boost external validity. The research also relied on respondent‐reported measurements, which could be manipulated by CMB and community desire systematic error. Though numerous procedures were implemented to mitigate such prejudices [67], their possible effect cannot be totally removed. Longitudinal approaches and data collection from different sources may provide better evidence in future studies. Furthermore, dependence on purposive sampling reduces external validity, which means that the outcomes may not be fully relevant to clinics other than the sample. As a consequence, we suggest that future research projects use probabilistic‐based sampling methods, including stratified or cluster sampling, and involve other stakeholders, including hospital directors, ecological officers, and medical personnel, to improve the accuracy and predictability of the findings. Lastly, this research looks at the links between GHRM, SWMP, EI, and SP in hospital contexts. However, it is critical to assess the possible impact of contextual and human parameters. Aspects including hospital size, nurse type, experience of nurses, hospital value system, and ecological policy can have a substantial influence on ecological outcomes. Larger medical facilities typically possess greater assets and technological ability to adopt garbage disposal and climate advances, whereas smaller medical facilities may experience resource limits that restrict the effect of GHRM and SWMP [29]. In addition, skilled nurses are more likely to comply with effective protocols, which makes them more experienced. On the other hand, newly appointed nurses are less skilled, and they need more training and mentorship for better service [38]. Organizational culture and working environment are serving as a supportive element to motivate workers to develop a sustainable mindset, but this area is not expressly explored in this work. Hospitals have to develop ISO‐1400 standard policy through promoting GHRM, SWMP, and EI in their operations. It is evident that GHRM, SWMP, and EI are significant for SP, but their success mainly depends on a sustainable‐oriented work environment and proper concern of HR. These areas are crucial for further research regarding this context.

## Author Contributions

Tipon Tanchangya and Kazi Omar Siddiqi drafted the original manuscript and conceptualized and designed the study method. Diponkor Chandra Das conceptualized and designed the study method. Farvez Islam and Abu Hena Md Martuza Ali collected, analyzed, and interpreted the data.

## Funding

The authors conducted this study without receiving any funds from different organizations, such as public, private, commercial, or NGOs.

## Ethics Statement

In terms of ethical issues, we took approval from the Ethical Review Committee (ERC) of Dhaka Medical College, Bangladesh (approval reference no: ERC‐DMC/ECC/2022/411). In addition, we ensure proper permission from the targeted health clinic, communicating with them formally and providing a questionnaire. We also add a context summary so that respondents can understand the study’s purpose, objectives, benefits, and potential risks. This study also respects participants’ consent and explores the objectives of this study. Furthermore, this study ensures data confidentiality. To ensure data accuracy and validity, a paragraph was added at the beginning of the questionnaire so that participants could understand the concept comprehensively. This study encourages participants to respond willingly, without any force.

## Conflicts of Interest

The authors declare no conflicts of interest.

## Data Availability

The datasets produced and examined in this study can be obtained from the corresponding author upon a reasonable request.

## References

[1] Farrukh M. , Xu S. , Baheer R. , and Ahmad W. , Unveiling the Role of Supply Chain Parameters Approved by Blockchain Technology Towards Firm Performance Through Trust: The Moderating Role of Government Support, Heliyon. (2023) 9, no. 11, 10.1016/j.heliyon.2023.e21831. PMC10663855 38028007

[2] Rahman M. A. , Moayedikia A. , and Wiil U. K. , Editorial: Data-Driven Technologies for Future Healthcare Systems, Frontiers Medical Technology. (2023) 5, 10.3389/fmedt.2023.1183687. PMC10244758 37293511

[3] Kanan M. , Taha B. , Saleh Y. et al., Green Innovation as a Mediator Between Green Human Resource Management Practices and Sustainable Performance in Palestinian Manufacturing Industries, Sustainability. (2023) 15, no. 2, 10.3390/su15021077.

[4] Ren G. , Jing M. , and Wang Z. , True Green or Fake Green? The Impact of ESG Rating Disagreement on Corporate Greenwashing Behavior, Business Ethics, the Environment & Responsibility. (2025) 10.1111/beer.70058.

[5] Sun J. , Sarfraz M. , Ivascu L. , and Ozturk I. , Unveiling Green Synergies: Sustainable Performance Through Human Resource Management, CSR, and Corporate Image Under a Mediated Moderation Framework, Environmental Science and Pollution Research. (2023) 30, no. 45, 101392–101409, 10.1007/s11356-023-29468-8. 37653190

[6] EPI , Environmental Performance Index, 2024, https://epi.yale.edu/downloads/2024-epi-report-20250106.pdf.

[7] Rahman M. M. , Islam M. F. , Dyuti T. I. , and Hossain M. E. , Driving Net Zero Emissions Through Green Finance, Green Logistics, and Corporate Social Responsibility: Findings From the PLS-SEM Approach, Discover Sustainability. (2024) 5, no. 1, 10.1007/s43621-024-00675-8.

[8] Dhaka Tribune , Hospital Waste Management in Bangladesh Lags Behind Sustainable Standards, 2025, https://www.dhakatribune.com/bangladesh/373802/hospital-waste-management-in-bangladesh-lags.

[9] Dihan M. R. , Nayeem S. M. A. , Roy H. et al., Healthcare Waste in Bangladesh: Current Status, the Impact of COVID-19 and Sustainable Management With Life Cycle and Circular Economy Framework, Science of the Total Environment. (2023) 871, 10.1016/j.scitotenv.2023.162083. PMC9908568 36764546

[10] Tushar S. R. , Moktadir M. A. , Kusi-Sarpong S. , and Ren J. , Driving Sustainable Healthcare Service Management in the Hospital Sector, Journal of Cleaner Production. (2023) 420, 10.1016/j.jclepro.2023.138310.

[11] Arslan L. and Dijkstra‐Silva S. , Effects of Combining Eco‐And Social Labels on Consumer Value—Additive, Neutral or Cannibalizing? Insights From a Conjoint Analysis, Business Ethics, the Environment & Responsibility. (2025) 1–16, 10.1111/beer.70039.

[12] Rana G. and Arya V. , Green Human Resource Management and Environmental Performance: Mediating Role of Green Innovation–A Study From an Emerging Country, Foresight. (2024) 26, no. 1, 35–58, 10.1108/fs-04-2021-0094.

[13] Zihan W. and Makhbul Z. K. M. , Green Human Resource Management as a Catalyst for Sustainable Performance: Unveiling the Role of Green Innovations, Sustainability. (2024) 16, no. 4, 10.3390/su16041453.

[14] Rice B. , Fieger P. , Martin N. , Raziq M. M. , and Rice J. , Exploring the Nexus of Organizational Culture and Corruption Reporting: Evidence From the Australian Public Service, Business Ethics, the Environment & Responsibility. (2025) 10.1111/beer.70064.

[15] Silva R. , Costa B. , and Martins F. , Environmental Impacts of Healthcare Waste Mismanagement and Sustainable Treatment Solutions, Journal of Environmental Management. (2024) 351, 10.1016/j.jenvman.2023.119788.

[16] Alrabiah H. , Ahmed V. , and Bahroun Z. , A Systematic Review of Waste Management Practices in the Healthcare Sector, Cleaner Waste Systems. (2025) 12, 10.1016/j.clwas.2025.100400.

[17] Tanchangya T. , Rahman J. , Siddiqi K. O. et al., Factors Affecting Green Banking Technology Adoption in Bangladesh, Discover Sustainability. (2025) 6, no. 1, 10.1007/s43621-025-02143-3.

[18] Health Bulletin , Government of the People’s Republic of Bangladesh: Ministry of Health and Family Welfare, 2023, https://dghs.portal.gov.bd/sites/default/files/files/dghs.portal.gov.bd/page/8983ee81_3668_4bc3_887e_c99645bbfce4/2024-10-30-15-17-04fcbbc3747cbf6cc6983a34770666d1.pdf.

[19] Zihan T. , Rahman M. , and Chowdhury S. , Green Innovation, Waste Management, and Sustainable Performance in Healthcare, Sustainable Operations and Computers. (2024) 5, 10.1016/j.susoc.2023.100240.

[20] Makumbe W. , Green Human Resources Management and Green Performance: A Mediation–Moderation Mechanism for Green Innovation and Green Knowledge Sharing, Sustainability. (2024) 16, no. 24, 10.3390/su162410849.

[21] Singh S. K. , Giudice M. , Chierici R. , and Graziano D. , Green Innovation and Environmental Performance: The Role of Green Transformational Leadership and Green Human Resource Management, Technological Forecasting and Social Change. (2020) 150, 10.1016/j.techfore.2019.119762.

[22] Seman N. A. A. , Govindan K. , Mardani A. et al., The Mediating Effect of Green Innovation on the Relationship Between Green Supply Chain Management and Environmental Performance, Journal of Cleaner Production. (2019) 229, 115–127, 10.1016/j.jclepro.2019.03.211.

[23] Hamed A. E. M. , Sefouhi L. , Ibrahim M. I. T. , Attia A. S. , Barakat A. M. , and Elsayed E. E. , From Knowledge to Impact: Revolutionizing Nursing Practices in Biomedical Waste Management for Sustainable Healthcare Excellence, BMC Nursing. (2025) 24, no. 1, 10.1186/s12912-025-03073-1. PMC12042339 40301813

[24] Khan M. A. and Kabir S. M. , Sustainable Waste Management and Organizational Performance in South Asian Healthcare Institutions, Environment, Development and Sustainability. (2023) 25, no. 8, 12345–12362, 10.1007/s10668-022-02271-9.

[25] Barney J. , Firm Resources and Sustained Competitive Advantage, Journal of Management. (1991) 17, no. 1, 99–120, 10.2307/259056.

[26] Appelbaum E. , Bailey T. , Berg P. , and Kalleberg A. L. , Manufacturing Advantage: Why High-Performance Work Systems Pay Off, 2000, Cornell University Press.

[27] Barney J. B. and Clark D. N. , Resource-Based Theory: Creating and Sustaining Competitive Advantage, 2007, Oxford University Press.

[28] Zhang X. , Xia N. , and Liu Y. , Sustainable Waste Management and Environmental Performance in Healthcare Institutions, Sustainability. (2021) 13, no. 1, 10.3390/su13010123.

[29] Correia A. B. , Farrukh M. , Moleiro M. J. , and Baheer R. , Impact of Green Human Resource Management Towards Sustainable Performance in the Healthcare Sector: Role of Green Innovation and Risk Management, Cogent Business & Management. (2024) 11, no. 1, 10.1080/23311975.2024.2374625.

[30] Jabbour C. J. C. , de Sousa Jabbour A. B. L. , Godinho Filho M. , and Roubaud D. , Environmental Management and Employee Empowerment: A Cleaner Production Perspective, Journal of Cleaner Production. (2020) 260, 10.1016/j.jclepro.2020.121031.

[31] Renwick D. W. , Redman T. , and Maguire S. , Green Human Resource Management: A Review and Research Agenda, International Journal of Management Reviews. (2013) 15, 1–14, 10.1111/j.1468-2370.2011.00328.x.

[32] Awan F. H. , Dunnan L. , Jamil K. et al., Mediating Role of Green Supply Chain Management Between Lean Manufacturing Practices and Sustainable Performance, Frontiers in Psychology. (2022) 12, 1–11, 10.3389/fpsyg.2021.810504. PMC8761733 35046878

[33] Mousa S. K. and Othman M. , The Impact of Green Human Resource Management Practices on Sustainable Performance in Healthcare Organisations: A Conceptual Framework, Journal of Cleaner Production. (2020) 243, 10.1016/j.jclepro.2019.118595.

[34] Janik-Karpinska E. , Brancaleoni R. , Niemcewicz M. et al., Healthcare Waste—A Serious Problem for Global Health, Healthcare. (2023) 11, no. 2, 10.3390/healthcare11020242. PMC9858835 36673610

[35] Das S. , Lee S. H. , Kumar P. , Kim K. H. , Lee S. S. , and Bhattacharya S. S. , Solid Waste Management: Scope and the Challenge of Sustainability, Journal of Cleaner Production. (2019) 2, no. 228, 658–678, 10.1016/j.jclepro.2019.04.323.

[36] Zamparas M. , Kapsalis V. C. , Kyriakopoulos G. L. et al., Medical Waste Management and Environmental Assessment in the Rio University Hospital, Western Greece, Sustainable Chemistry and Pharmacy. (2019) 3, no. 2, 124–135, 10.1016/j.scp.2019.100163.

[37] Silva T. , Santos Â. R. S. , Maciel R. , Santos S. M. , and Florencio L. , Use of the Value-Focused Thinking Methodology to Understand Health Care Waste Management Under the Perspective of Occupational and Environmental Health, Journal of Cleaner Production. (2024) 479, 10.1016/j.jclepro.2024.144036.

[38] Pham V. K. , Vu T. N. Q. , Phan T. T. , and Nguyen N. A. , The Impact of Organizational Culture on Employee Performance: A Case Study at Foreign-Invested Logistics Service Enterprises Approaching Sustainability Development, Sustainability. (2024) 16, no. 15, 10.3390/su16156366.

[39] Niazi U. I. , Nisar Q. A. , Nasir N. , Naz S. , Haider S. , and Khan W. , Green HRM, Green Innovation and Environmental Performance: The Role of Green Transformational Leadership and Green Corporate Social Responsibility, Environmental Science and Pollution Research. (2023) 30, no. 15, 45353–45368, 10.1007/s11356-023-25442-6. 36705831

[40] Soewarno N. , Tjahjadi B. , and Fithrianti F. , Green Innovation Strategy and Green Innovation: The Roles of Green Organizational Identity and Environmental Organizational Legitimacy, Management Decision. (2019) 57, no. 11, 3061–3078, 10.1108/MD-05-2018-0563.

[41] Shipton H. , Sparrow P. , Budhwar P. , and Brown A. , HRM and Innovation: Looking Across Levels, Human Resource Management Journal. (2017) 27, no. 2, 246–263, 10.1111/1748-8583.12102.

[42] Ghasemi M. K. , Development of a Sustainable Healthcare Waste Management Model Using a Hybrid Multiple Decision-Making Model, 2018, Universiti Putra Malaysia.

[43] Sherman J. D. and Singh H. , Bringing Environmental Sustainability Into the Quality Agenda: Time to Act on Reducing Health Care Pollution and Waste, Joint Commission Journal on Quality and Patient Safety. (2023) 49, no. 6, 336–339, 10.1016/j.jcjq.2023.02.006. 37024422

[44] Asadi S. , Pourhashemi S. , Nilashi M. et al., Investigating Influence of Green Innovation on Sustainability Performance: A Case on Malaysian Hotel Industry, Journal of Cleaner Production. (2020) 258, 10.1016/j.jclepro.2020.120860.

[45] Khan W. , Nisar Q. A. , Roomi M. A. , Nasir S. , Awan U. , and Rafiq M. , Green Human Resources Management, Green Innovation and Circular Economy Performance: The Role of Big Data Analytics and Data-Driven Culture, Journal of Environmental Planning and Management. (2024) 67, no. 10, 2356–2381, 10.1080/09640568.2023.2189544.

[46] Awan F.H. , Dunnan L. , Jamil K. , and Gul R.F. , Stimulating Environmental Performance via Green Human Resource Management, Green Transformational Leadership, and Green Innovation: A Mediation-Moderation Model, Environmental Science and Pollution Research. (2023) 30, no. 2, 2958–2976, 10.1007/s11356-022-22424-y. 35939187

[47] Mertler C. A. , Introduction to Educational Research, 2022, Sage.

[48] Rozario M. D. , Adhikary H. , Gazi H. R. , and Begum D. , Nurses’ Roles in Patient Care in Tertiary Level Hospitals in Bangladesh, Bangladesh Medical Research Council Bulletin. (2018) 44, no. 3, 138–144, 10.3329/bmrcb.v44i3.39937.

[49] Wolf E. J. , Harrington K. M. , Clark S. L. , and Miller M. W. , Sample Size Requirements for Structural Equation Models an Evaluation of Power, Bias, and Solution Propriety, Educational and Psychological Measurement. (2013) 73, no. 6, 913–934, 10.1177/0013164413495237. PMC4334479 25705052

[50] Facility Registry (Government of People’s Republic of Bangladesh, Ministry of Health and Family Welfare) , HRM Status, 2025, Dhaka Medical College Hospital, https://hris.mohfw.gov.bd/public/facility-registry/facilities/31/profile?tab=hrm-status.

[51] Facility Registry (Government of People’s Republic of Bangladesh, Ministry of Health and Family Welfare) , HRM Status, 2025, Cumilla Medical College Hospital, https://hrm.dghs.gov.bd/public/facility-registry/facilities/862/profile?tab=hrm-status.

[52] Facility Registry (Government of People’s Republic of Bangladesh, Ministry of Health and Family Welfare) , HRM Status, 2025, Mymensingh Medical College Hospital, https://hris.mohfw.gov.bd/public/facility-registry/facilities/392/profile?tab=hrm-status.

[53] Roy A. , Van Der Weijden T. , and De Vries N. , Relationships of Work Characteristics to Job Satisfaction, Turnover Intention, and Burnout Among Doctors in the District Public-Private Mixed Health System of Bangladesh, BMC Health Services Research. (2017) 17, no. 1, 1–11, 10.1186/s12913-017-2369-y. 28637454 PMC5480190

[54] Polit D. F. and Beck C. T. , Nursing Research: Generating and Assessing Evidence for Nursing Practice, 2017, 10th edition, Wolters Kluwer.

[55] Althubaiti A. , Information Bias in Health Research: Definition, Pitfalls, and Adjustment Methods, Journal of Multidisciplinary Healthcare. (2016) 9, 211–217, 10.2147/JMDH.S104807. 27217764 PMC4862344

[56] Kock N. , Common Method Bias in PLS-SEM: A Full Collinearity Assessment Approach, International Journal of e-Collaboration. (2015) 11, no. 4, 1–10, 10.4018/ijec.2015100101.

[57] Siddiqi K. O. , Rahman M. H. , Esquivias M. A. , and Hutapea L. M. N. , The Effect of Perceived Organizational and Supervisor Support on Nurses’ Turnover Intention in Bangladesh: Does Work-Family Conflict Play a Role?, Social Sciences & Humanities Open. (2024) 10, 10.1016/j.ssaho.2024.100992.

[58] Liao H. , Liu D. , and Loi R. , Looking at Both Sides of the Social Exchange Coin: A Social Cognitive Perspective on the Joint Effects of Relationship Quality and Differentiation on Creativity, Academy of Management Journal. (2010) 53, no. 5, 1090–1109, 10.5465/amj.2010.54533207.

[59] Hair J. F. , Black W. C. , Babin B. J. , and Anderson R. E. , Multivariate Data Analysis, 2019, 8th edition, Cenage Learning.

[60] Dumont J. , Shen J. , and Deng X. , Effects of Green HRM Practices on Employee Workplace Green Behavior: The Role of Psychological Green Climate and Employee Green Values, Human Resource Management. (2017) 56, no. 4, 613–627, 10.1002/hrm.21792.

[61] Quartey E. K. , Nyamah E. Y. , and Charnor I. T. , Waste Management Practices and Environmental Sustainability Performance: Does Institutional Pressure Matter?, Journal of Responsible Production and Consumption. (2025) 2, no. 1, 345–374, 10.1108/JRPC-11-2024-0063.

[62] Paille P. , Chen Y. , Boiral O. , and Jin J. , The Impact of Human Resource Management on Environmental Performance: An Employee Study, Journal of Business Ethics. (2014) 121, no. 3, 451–466, 10.1007/s10551-013-1732-0.

[63] Wong C. Y. , Wong C. W. , and Boon-itt S. , Effects of Green Supply Chain Integration and Green Innovation on Environmental and Cost Performance, International Journal of Production Research. (2020) 58, no. 15, 4589–4609, 10.1080/00207543.2020.1756510.

[64] Dash G. and Paul J. , CB-SEM Versus PLS-SEM Methods for Research in Social Sciences and Technology Forecasting, Technological Forecasting and Social Change. (2021) 173, 10.1016/j.techfore.2021.121092.

[65] Dul J. , Identifying Single Necessary Conditions With NCA and fsQCA, Journal of Business Research. (2016) 69, no. 4, 1516–1523, 10.1016/j.jbusres.2015.10.134.

[66] Ho R. , Handbook of Univariate and Multivariate Data Analysis and Interpretation With SPSS, 2006, 1st edition, Chapman and Hall/CRC.

[67] Hair J. F.Jr., Hult G. T. M. , Ringle C. M. , and Sarstedt M. , A Primer on Partial Least Squares Structural Equation Modeling (PLS-SEM), 2022, 3rd edition, Sage.

[68] Fornell C. and Larcker D. F. , Evaluating Structural Equation Models With Unobservable Variables and Measurement Error, Journal of Marketing Research. (1981) 18, no. 1, 39–50, 10.2307/3151312.

[69] Henseler J. , Hubona G. S. , and Ray P. A. , Using PLS Path Modeling in New Technology Research: Updated Guidelines, Industrial Management and Data Systems. (2016) 116, no. 1, 1–19, 10.1108/IMDS-09-2015-0382.

[70] Hair J. F. , Howard M. C. , and Nitzl C. , Assessing Measurement Model Quality in PLS-SEM Using Confirmatory Composite Analysis, Journal of Business Research. (2020) 109, 101–110, 10.1016/j.jbusres.2019.11.069.

[71] Singh R. , Does My Structural Model Represent the Real Phenomenon? A Review of the Appropriate Use of Structural Equation Modelling Model Fit Indices, The Marketing Review. (2009) 9, no. 3, 199–212, 10.1362/146934709X467767.

[72] Dul J. , Van der Laan E. , and Kuik R. , A Statistical Significance Test for Necessary Condition Analysis, Organizational Research Methods. (2020) 23, no. 2, 385–395, 10.1177/1094428118795272.

[73] Dul J. , Vis B. , and Goertz G. , Necessary Condition Analysis (NCA) Does Exactly What It Should Do When Applied Properly: A Reply to a Comment on NCA, Sociological Methods & Research. (2021) 50, no. 2, 926–936, 10.1177/0049124118799383.

[74] Aftab J. , Abid N. , Cucari N. , and Savastano M. , Green Human Resource Management and Environmental Performance: The Role of Green Innovation and Environmental Strategy in a Developing Country, Business Strategy and the Environment. (2023) 32, no. 4, 1782–1798, 10.1002/bse.3219.

[75] Jnaneswar K. , Green HRM and Employee Green Behavior Min the Manufacturing Firms: Do Psychological Green Climate and Employee Green Commitment Matter?, Social Responsibility Journal. (2024) 19, no. 10, 1852–1869, 10.1108/SRJ-11-2022-0477.

[76] Hooi L. W. , Liu M. , and Lin J. J. J. , Green Human Resource Management and Green Organizational Citizenship Behavior: Do Green Culture and Green Values Matter?, International Journal of Manpower. (2022) 43, no. 3, 763–785, 10.1108/IJM-05-2020-0247.

[77] Khan S. A. R. , Zhang Y. , Kumar A. , Zavadskas E. , and Streimikiene D. , Measuring the Impact of Waste Management on Sustainable Development: A System Dynamics Approach, Journal of Cleaner Production. (2020) 242, 10.1016/j.jclepro.2019.118450.

[78] Riaz A. , Al-Okaily M. , Sohail A. , Ashfaq K. , and Rehman S. U. , Green Human Resource Management and Sustainable Performance: Serial Mediating Role of Green Knowledge Management and Green Innovation, Global Knowledge, Memory and Communication (ahead-of-print). (2024) 10.1108/GKMC-03-2024-0127.

[79] Yong J. Y. , Yusliza M. Y. , Ramayah T. , and Fawehinmi O. , Nexus Between Green Intellectual Capital and Green Human Resource Management, Journal of Cleaner Production. (2020) 215, 364–374, 10.1016/j.jclepro.2018.12.306.

[80] Liu D. , Yousaf Z. , and Rosak-Szyrocka J. , Environmental Performance Through Green Supply Chain Management Practices, Green Innovation, and Zero Waste Management, Sustainability. (2024) 16, no. 24, 10.3390/su162411173.

[81] AlSheyadi A. , Abbas H. , Baawain A. , and Saeed A. , Linking Zero-Waste Management and Green Innovative Supply Chains to Sustainable Performance: The Mediating Role of Green Dynamic Capabilities in Manufacturing Firms, Sustainability. (2025) 17, no. 22, 10.3390/su172210348.

[82] Lee J. W. , Kim Y. M. , and Kim Y. E. , Antecedents of Adopting Corporate Environmental Responsibility and Green Practices, Journal of Business Ethics. (2018) 148, no. 2, 397–409, 10.1007/s10551-016-3024-y.

[83] Jackson S. E. and Seo J. , The Greening of Strategic HRM Scholarship, Organization Management Journal. (2010) 7, no. 4, 278–290, 10.1057/omj.2010.37.

[84] Wright C. and Nyberg D. , An Inconvenient Truth: How Organizations Translate Climate Change Into Business as Usual, Academy of Management Journal. (2017) 60, no. 5, 1633–1661, 10.5465/amj.2015.0718.

[85] Kraus S. , Rehman S. U. , and García F. J. S. , Corporate Social Responsibility and Environmental Performance: The Mediating Role of Environmental Strategy and Green Innovation, Technological Forecasting and Social Change. (2020) 160, 10.1016/j.techfore.2020.120262.

[86] DeLisi C. , Patrinos A. , MacCracken M. et al., The Role of Synthetic Biology in Atmospheric Greenhouse Gas Reduction: Prospects and Challenges, BioDesign Research. (2018) 2020, 10.34133/2020/1016207. PMC10521736 37849905

[87] World Health Organization (WHO) , Safe Management of Wastes From Health-Care Activities, 2018, 2nd edition, WHO, https://www.who.int/publications/i/item/9789241514705.

[88] Rahman M. A. , Hossain M. A. , and Chowdhury F. , Healthcare Sustainability and Environmental Governance in Bangladesh, Sustainability. (2021) 13, no. 14, 10.3390/su13147800.

