# Correction to “Leveraging Nursing Management for Sustainable Healthcare: The Synergistic Impact of Green Human Resource Management, Sustainable Waste Management Practices, and Environmental Innovation”

**DOI:** 10.1155/jonm/9836031

**Published:** 2026-08-27

T. Tanchangya, D. C. Das, F. Islam, A. H. M. Ali, & K. O. Siddiqi, Leveraging Nursing Management for Sustainable Healthcare: The Synergistic Impact of Green Human Resource Management, Sustainable Waste Management Practices, and Environmental Innovation. *Journal of Nursing Management*. 2026; 2026:3381860. https://doi.org/10.1155/jonm/3381860.

In the “Methods—3.1 Sample and Procedure” section, the text:

“In Bangladesh, Roy et al. [53] found that using the existing list of nurses and applying the random sampling method is not feasible for the following reasons: most of the nurses change their workplace very often, and those nurses who work in public hospitals also work in private hospitals part‐time. Therefore, using random sampling might result in duplicating public healthcare workers.”

was incorrect. This should have read:

“In Bangladesh, Roy et al. [53] reported that using existing lists of doctors and applying random sampling was not feasible because many doctors frequently changed their workplaces and some worked simultaneously in both public and private hospitals, which could result in duplication within the sampling frame. A similar situation applies to nurses in Bangladesh, as nurses may also change workplaces and, in some cases, work across both public and private healthcare facilities. Therefore, consistent with the rationale provided by Roy et al. [53], the present study adopted a non‐probabilistic purposive sampling approach to recruit eligible nurses.”

We apologize for this error.

